# A focus on detection of polymorphs by dynamic nuclear polarization solid-state nuclear magnetic resonance spectroscopy

**DOI:** 10.1039/d3sc90177g

**Published:** 2023-09-28

**Authors:** Yunhua Chen, Jiashan Mi, Aaron J. Rossini

**Affiliations:** a Department of Chemistry, Iowa State University Ames IA 50011 USA arossini@iastate.edu; b Analytical Research & Development, AbbVie, Inc. North Chicago Illinois 60064 USA

## Abstract

Solid-state nuclear magnetic resonance (ssNMR) spectroscopy has found increasing application as a method for quantification and structure determination of solid forms (polymorphs) of organic solids and active pharmaceutical ingredients (APIs). However, ssNMR spectroscopy suffers from low sensitivity and resolution, making it challenging to detect dilute solid forms that may be present after recrystallization or reaction with co-formers. Cousin *et al.* (S. F. Cousin *et al.*, *Chem. Sci.*, 2023, https://doi.org/10.1039/D3SC02063K) have demonstrated that dynamic nuclear polarization (DNP) enhanced ^13^C cross-polarization (CP) saturation recovery experiments can be used to detect dilute polymorphic forms that are present within a mixture of solid forms. Enhancement of the NMR signal by DNP and differences in signal build-up rates for different polymorphs provide the sensitivity and contrast needed to resolve NMR signals from minor polymorphic forms. This method demonstrated by Cousin *et al.* should aid the discovery of solid drug forms.

## Abstract 2

Cousin *et al.* (*Chem. Sci.*, 2023, https://doi.org/10.1039/D3SC02063K) have demonstrated that dynamic nuclear polarization ^13^C solid-state nuclear magnetic resonance spectroscopy can be used to detect dilute polymorphic forms that are present within a mixture of solid forms.

## Graphical abstract

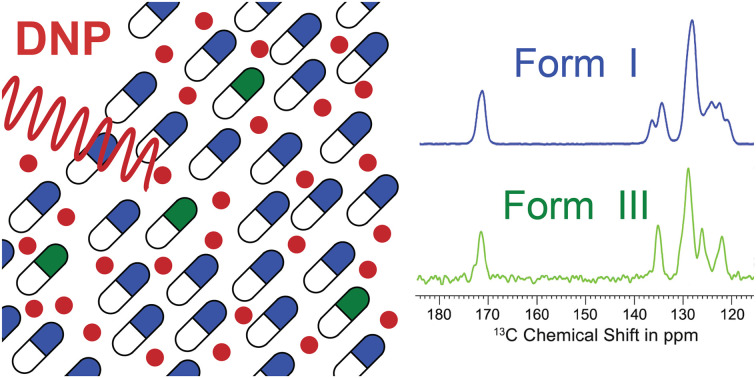

Different solid phases (forms) of active pharmaceutical ingredients (APIs) display varying stability, solubility, and bioavailability and can also be patented.^1^ Consequently, when solid APIs are being prepared for formulation, crystallization screening experiments are used to search for as many solid phases as possible.^1^ However, even after extensive solid form screening, new crystal forms can be discovered.^2,3^ The unexpected emergence of API forms can pose challenges, particularly during late-stage product development or post-launch, as newfound API forms may exhibit undesirable properties, such as reduced solubility.^2,3^ Diffraction, microscopy, and spectroscopy techniques are used to structurally characterize and detect different solid drug forms.^4,5^ Solid-state nuclear magnetic resonance (ssNMR) spectroscopy is a powerful technique for polymorph characterization because it can probe the 3D arrangement of atoms and their motions *via* measurements of chemical shifts, coupling constants, and relaxation times.^6–9^ However, ssNMR spectroscopy generally suffers from poor sensitivity, meaning it is challenging (and sometimes impossible) to detect minor API phases within mixtures or dilute APIs in drug formulations.

Due to the efforts of Griffin and co-workers, dynamic nuclear polarization (DNP) has emerged as a method to routinely enhance the sensitivity of magic angle spinning (MAS) ssNMR experiments.^10^ In a DNP experiment the sample is doped with stable free radicals. Microwave irradiation at or near to the electron Larmor frequency is used to facilitate polarization transfer from electron spins to the surrounding nuclear spins, typically resulting in a 1–2 order of magnitude improvement in NMR sensitivity. Relayed DNP experiments have previously been used to enhance the sensitivity of 1D and 2D ssNMR experiments on organic solids, pure APIs, and formulated APIs.^11^ In a relayed DNP experiment on crystalline APIs, the radicals will be restricted to the surface of the material, resulting in an initial build-up of ^1^H polarization at the surface of the crystals.^12^ However, ^1^H nuclear spin diffusion will spontaneously relay polarization from surface ^1^H spins to sub-surface or bulk ^1^H spins.^12–14^ Polarization from the ^1^H spins can then be transferred to nearby heteronuclear spins such as ^13^C, ^15^N, ^17^O, *etc.* using pulsed NMR methods. The efficiency of the ^1^H nuclear spin diffusion process and DNP enhancements are determined by the particle size distribution, the concentration of ^1^H spins and ^1^H *T*_1_.^12,13^

The recent work of Cousin *et al.* “Exploiting solid-state dynamic nuclear polarization NMR spectroscopy to establish the spatial distribution of polymorphic phases in a solid material” uses the features of relayed DNP experiments to detect minor polymorphic impurity phases present at a concentration of a few weight percent.^15^ In this paper they studied crystallized samples of *meta*-aminobenzoic acid (*m*-ABA) because this is a well-studied model system known to exhibit polymorphism. Most of the *m*-ABA they studied existed as Form I, however, there was a small amount of Form III *m*-ABA that was naturally present after recrystallization. DNP-enhanced ^1^H–^13^C CPMAS saturation recovery experiments were used to monitor the build-up of ^1^H polarization in the *m*-ABA samples as a function of the DNP polarization delay (*τ*, Fig. 1a). As expected, the 1D ^13^C ssNMR spectra only show NMR signals from the more concentrated Form I because the Form III NMR signals are much weaker and are obscured by those of Form I (Fig. 1b and c). However, subtraction of the DNP-enhanced ^13^C ssNMR spectrum recorded with *τ* of 12 s from the spectrum recorded with *τ* of 350 s revealed the ^13^C NMR spectrum of Form III (Fig. 1d). Form III had a much longer ^1^H DNP build-up time than Form I, allowing the two different drug forms to be resolved in the saturation recovery experiment. The results shown in Fig. 1 are significant as they demonstrate the potential utility of DNP-enhanced ^13^C ssNMR in the context of solid form screening. The DNP enhancements and signal build-up rates provide the contrast needed to resolve NMR signals from minor polymorphic forms, while the signal enhancement provided by DNP enables the detection of the NMR spectrum of the dilute solid form in the mixture. Furthermore, the authors used numerical simulations of ^1^H spin diffusion to model DNP build-up curves for different micron-scale spatial distributions of Form I and Form III. The authors’ simulations suggest that spatial segregation of Form I and Form III crystals best models the NMR experiments (Model B, Fig. 2). Overall, the experiments of Cousin *et al.* illustrate many potential advantages of DNP-enhanced ssNMR spectroscopy for investigating crystallization of APIs.

**Fig. 1:**
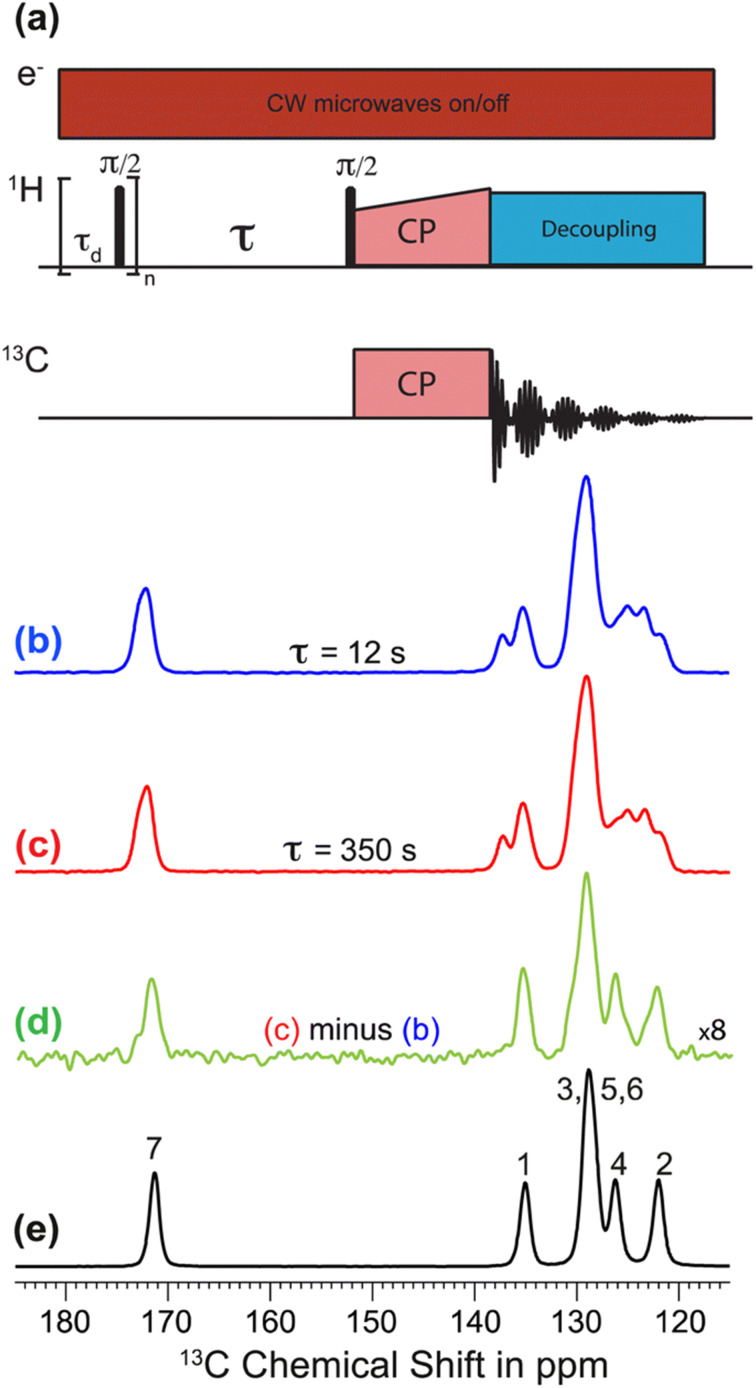
(a) ^1^H–^13^C CPMAS saturation–recovery pulse sequence used to record solid-state ^13^C NMR spectra for different polarization times (*τ*). (b and c) DNP-enhanced solid-state ^13^C NMR spectra of powdered *m*-ABA recorded at 110 K with (b) *τ* = 12 s and (c) *τ* = 350 s. (d) Difference spectrum. (e) Solid-state ^13^C NMR spectrum recorded at 110 K for a sample of pure Form III *m*-ABA. Reproduced with permission from ref. 15.

**Fig. 2:**
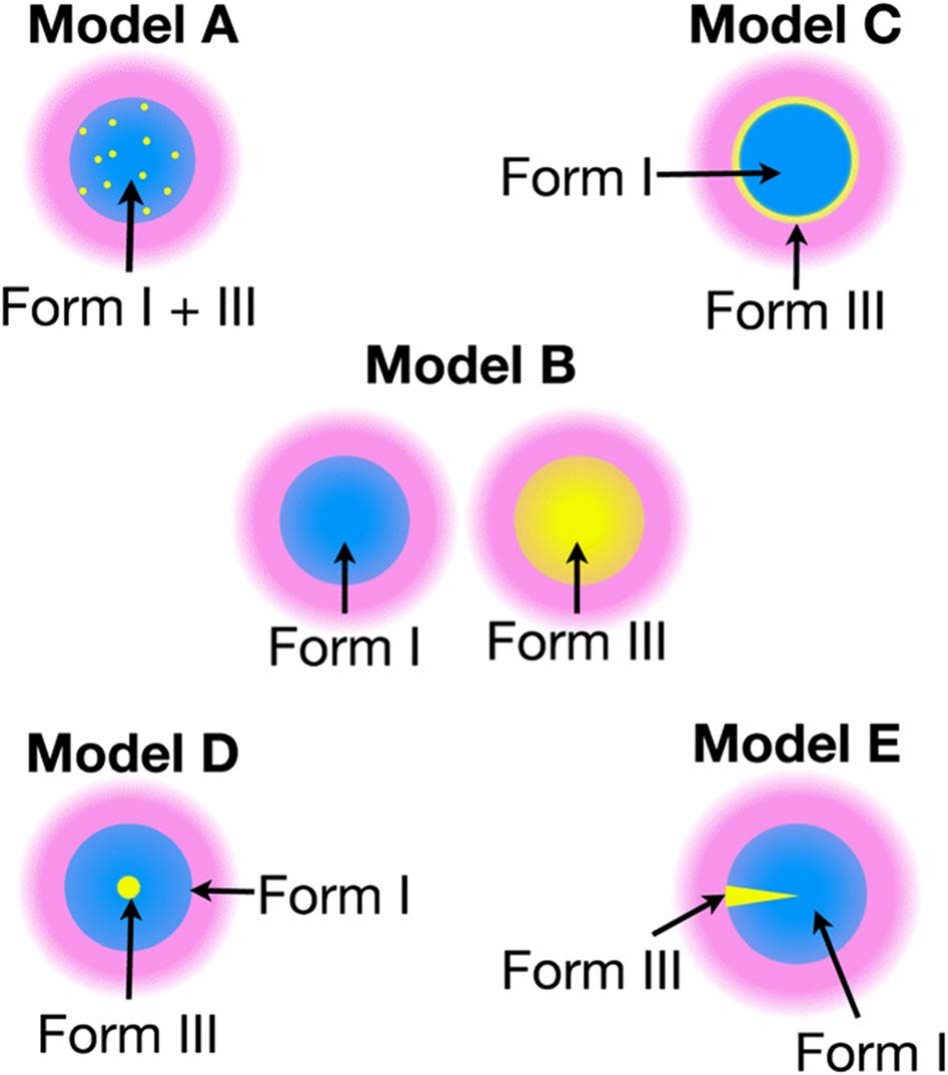
Models A–E show different possible distributions of the two *m*-ABA polymorphs, Form I and Form III. The solution (pink) containing the polarizing agent is located on the surface of the particles. Numerical simulations of ^1^H spin diffusion suggested that model B best reproduced the DNP saturation recovery experiments. Reproduced with permission from ref. 15.

There are exciting future directions for these types of NMR experiments, with the most obvious being a better integration with theoretical methods. Computational crystal structure prediction (CSP) has grown increasingly powerful in the past few years, enabling prediction of possible crystal forms and ranking of their lattice energies.^16^ Relatedly, ^1^H and ^13^C chemical shifts can be accurately calculated for different crystal forms using planewave DFT^17^ or machine-learning based methods.^18^ The combinations of experimental chemical shift measurement, CSP and calculations of NMR chemical shifts has been dubbed “NMR crystallography” as it enables crystal structure determination from NMR observables.^19,20^ Therefore, the ability to observe the NMR spectrum of a crystalline organic solid provides the potential means to determine its crystal structure. Using the experiments demonstrated by Cousin *et al.* and CSP it should be possible to record the NMR signatures of dilute crystal forms present in a mixture, determine their crystal structures and assess if they are worthwhile targets for further development and exploration. Finally, characterization of the spatial distribution of the different crystalline forms by DNP could provide insight into ways to process solid APIs to remove undesired crystal forms or to discover new solid phases.

## Author contributions

Y. C., J. M. and A. J. R. equally contributed to writing the article.

## Conflicts of interest

There are no conflicts to declare.

## Supplementary Material
